# Replicating vs. non-replicating RNA therapeutics: Mechanisms, trade-offs, and future directions

**DOI:** 10.1016/j.omtn.2026.103153

**Published:** 2026-10-21

**Authors:** Min-Hsuan Lin, David Harrich

**Affiliations:** 1 Program of Infection and Inflammation, QIMR Berghofer Medical Research Institute, Herston, QLD 4006, Australia; 2 Global Virus Network Center of Excellence, Australian Infectious Disease Research Centre, St Lucia, QLD 4067, Australia; 3 Centre for Tropical Health and Emerging Diseases, Herston, QLD 4006, Australia

**Keywords:** MT: RNA/DNA editing, RNA therapeutics, replicating RNA, non-replicating RNA, self-amplifying RNA, defective interfering RNA, defective viral genome, immunity, antiviral Therapy, RNA delivery

## Abstract

RNA-based therapies are providing new ways to treat infections, cancer, and inherited diseases. These therapies fall into two broad categories: non-replicating RNAs, like messenger RNA, small interfering RNA (siRNA), and antisense oligonucleotides; and replicating RNAs, such as self-amplifying RNA (saRNA) and defective interfering (DI) RNAs. Non-replicating RNAs act in a single round and depend on precise chemical design and effective delivery. Replicating RNAs make more copies of themselves inside cells directly or conditionally, which can improve their strength, duration, and ability to trigger immune responses. In this review, we compare how these two groups of RNA therapies work, how they are designed and delivered, and where they are best suited. We discuss differences in immune effects, dosing, and manufacturing, and include long-studied DI RNAs, now being developed as a new type of antiviral therapy with broad activity and a favorable emerging preclinical safety profile. We also explore delivery methods such as lipid nanoparticles (LNPs) and polymer-based systems. By comparing replicating and non-replicating strategies, we aim to clarify their roles in RNA medicine and highlight how they might be combined in future treatments.

## Graphical abstract

**Figure:**
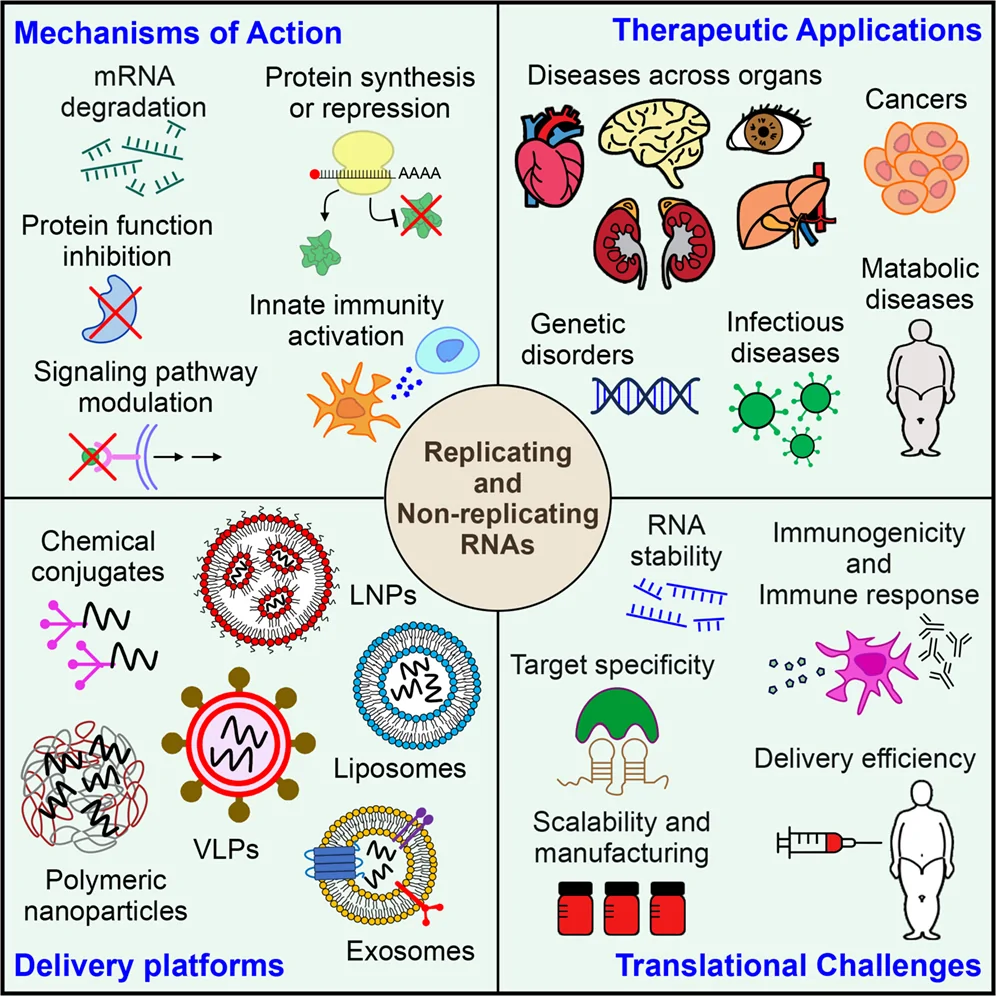

## Teaser

Lin and Harrich compare replicating and non-replicating RNA platforms, highlighting how replication capacity shapes potency, durability, immune activation, delivery, safety, and manufacturing. Emerging approaches, including self-amplifying RNAs, viral replicons, and defective interfering RNAs, illustrate how these properties can be harnessed to guide the design of next-generation RNA medicines.

## Introduction

The concept of using RNA as a therapeutic agent has evolved dramatically over the past three decades, transitioning from a scientific curiosity to a clinical reality. Early efforts focused on antisense oligonucleotides (ASOs) and RNA interference (RNAi), culminating in the first small interfering RNA (siRNA)-based drug approval in 2018 with the US Food and Drug Administration (FDA) approval of patisiran (Onpattro) for hereditary transthyretin-mediated amyloidosis.^1^ The subsequent development of chemically modified messenger RNA (mRNA)-enabled transient protein expression with improved stability and reduced immunogenicity, paving the way for the rapid development and scalable manufacture of mRNA vaccines during the COVID-19 pandemic.^2^^,^^3^^,^^4^^,^^5^ Parallel to these advances, defective interfering (DI) RNAs, naturally occurring viral genomes with large internal deletions, have gained renewed interest as conditionally replication-competent RNA molecules with dual antiviral and immunostimulatory activity.^6^^,^^7^ Together, these platforms illustrate the versatility of RNA as both a drug and a drug-encoding molecule. As the field matures, it is increasingly clear that RNA therapeutics can be categorized based on their capacity for intracellular replication, a feature that fundamentally shapes their pharmacodynamics, safety profiles, and delivery requirements. A recent review by Androsavich et al. provides a comprehensive framework that unifies lessons learned from across RNA therapeutic classes, emphasizing convergent design principles and shared challenges across siRNAs, ASOs, mRNAs, and self-amplifying RNAs (saRNAs).^8^

Given journal limitations on the number of citations permitted, references have been selected to be representative rather than exhaustive; we apologize to authors whose work could not be cited.

As the diversity of RNA-based therapeutics expands, a unifying framework is needed to compare their functional properties, mechanisms, and translational potential. One of the most informative distinctions is whether a therapeutic RNA can replicate within host cells. Replication competency fundamentally alters the pharmacokinetics and biological effects of the RNA: non-replicating RNAs typically require chemical modification, higher doses, or repeat administration to sustain activity, whereas replicating RNAs can amplify intracellularly, prolonging expression and enhancing potency from a single dose. It should be noted, however, that duration of action is not synonymous with replication competency: chemically modified siRNAs and ASOs can achieve months-long target suppression through *N*-acetylgalactosamine (GalNAc) conjugation, subcellular sequestration, or tissue accumulation, independently of any intracellular amplification.^9^^,^^10^ Replication competency therefore defines a mechanistic axis (intracellular RNA amplification), rather than a pharmacokinetic one, and the two-category framework used here reflects this molecular distinction. This intracellular amplification can also influence innate immune activation: replication generates double-stranded RNA intermediates that are recognized by cytoplasmic retinoic acid-inducible gene I (RIG-I)-like receptors (RLRs), including RIG-I and melanoma differentiation-associated protein 5 (MDA5).^11^ Importantly, however, alphaviral replication proceeds within membrane-bound spherules that partially sequester these intermediates from cytosolic sensors, so replication per se does not necessarily generate greater innate stimulation than a non-replicating RNA of comparable formulation and purity.^12^ Furthermore, the choice between a replicating or non-replicating platform affects formulation strategies, delivery routes, safety profiles, and regulatory considerations. Categorizing RNA therapeutics along this axis allows for clearer comparison of existing technologies and more rational design of future RNA medicines based on therapeutic context and clinical need.

This review aims to provide a comprehensive comparison of replicating and non-replicating RNA therapeutics, with a focus on their molecular mechanisms, therapeutic applications, delivery strategies, and translational challenges. By examining well-established platforms such as siRNAs, mRNAs, and ASOs alongside emerging replicating RNA modalities like saRNAs and DI RNAs, we highlight the unique strengths and limitations inherent to each approach. Particular emphasis is placed on antiviral applications, where replication-competent RNAs offer advantages in potency, duration, and immune activation. We also address critical trade-offs, such as dosing requirements, immunogenicity, and manufacturing complexity, that influence platform selection. Through this framework, we aim to guide the rational development of RNA therapeutics and identify opportunities for innovation, including hybrid designs that leverage the best features of both replicating and non-replicating systems.

## Non-replicating RNA therapeutics

Non-replicating RNA therapeutics encompass a broad range of modalities that exert their effects without intracellular RNA amplification. These molecules, ranging from mRNAs to siRNAs, ASOs, aptamers, decoys, and microRNA (miRNA)-targeting agents, act through single-round engagement with host cell machinery and precise regulation of gene expression or protein output. Their pharmacologic function relies on delivery to the appropriate cellular compartment, followed by translation, hybridization, or structural binding. Because they do not encode viral replicase or generate RNA progeny, non-replicating RNAs require chemical stabilization to sustain activity; for some modalities such as mRNA, this necessitates repeated dosing, whereas chemically modified siRNAs and ASOs can achieve durable target suppression lasting months to years through tissue accumulation and high target affinity. These strong advantages in safety, predictability, and regulatory maturity have positioned non-replicating RNAs at the forefront of approved RNA medicines. Table 1 describes the major types of non-replicating RNAs used to treat diseases, including their delivery platforms, features, and associated challenges.

**Table 1:** Non-replicating RNA therapeutics: Delivery strategies, mechanisms, and key considerations

RNA type	Common delivery platforms	Typical or explored routes	Notable features	Delivery challenges	Duration of action	References
mRNA	• LNPs • polymeric nanoparticles	commonly intramuscular for vaccines intravenous or local delivery for some therapeutic applications	• transient protein expression • scalable manufacturing	• degradation by nucleases • endosomal escape	transient (days to weeks)	Polack,^4^ Baden,^5^ Swingle et al.^13^
siRNA	• GalNAc conjugates • LNPs • polymeric carriers	usually subcutaneous for GalNAc-mediated hepatocyte targeting intravenous for LNP-formulated siRNAs	• sequence-specific gene silencing • liver targeting via GalNAc	• poor extrahepatic delivery • rapid clearance	moderate to durable (weeks to months; up to ∼6 months with GalNAc conjugates)	Springer and Dowdy^14^
ASO	• naked delivery • GalNAc conjugates • intrathecal injection	depending on target tissue: subcutaneous, intravenous, intrathecal, or local injection	• splicing modulation or RNase H activation • approved CNS delivery	• low cellular uptake • need for repeated dosing	durable (months to years; annual dosing for some approved drugs)	Lauffer et al.^15^
Aptamer/RNA Decoy	• PEGylation • nanoparticle carriers	intraocular for approved aptamer use intravenous or target-site injection depending on target and formulation	• high-specificity binding • potential antibody alternatives	• rapid renal clearance • nuclease sensitivity	moderate (days to weeks; dependent on chemical modification)	Ni et al.,^16^ Bege et al.^17^
miRNA mimic/inhibitor	• LNPs • chemically modified oligonucleotides	intravenous or local delivery. route remains platform- and indication-dependent.	• modulate endogenous regulatory networks	• off-target effects • tissue-specific delivery	moderate (weeks to months)	Matsui and Corey,^18^ Liang and He,^19^ Diener et al.^20^

LNPs, lipid nanoparticles; GalNAc, *N*-acetylgalactosamine; ASO, antisense oligonucleotide; CNS, central nervous system.

### Messenger RNA therapeutics

mRNA therapeutics deliver synthetic transcripts that are translated by the host’s cellular machinery into functional proteins. Their speed of design, platform versatility, and transient expression profile make mRNA attractive for both prophylactic and therapeutic applications. The global rollout of mRNA vaccines by Moderna and BioNTech-Pfizer during the COVID-19 pandemic provided proof-of-concept for scalable mRNA-based immunization, demonstrating robust immunogenicity, rapid manufacturing, and acceptable safety when delivered via lipid nanoparticles (LNPs).^4^^,^^5^

Beyond vaccines, mRNA therapeutics are being developed in preclinical and clinical studies for protein replacement in genetic diseases such as cystic fibrosis, ornithine transcarbamylase deficiency, and methylmalonic acidemia. Moderna’s mRNA-3705 for methylmalonic acidemia has advanced to phase 1/2 clinical evaluation (NCT05295433), as has mRNA-3927 for propionic acidemia.^21^ To our knowledge, as of June 1, 2026, no non-vaccine mRNA therapy has received full regulatory approval; however, multiple candidates, including mRNA-based protein replacement therapies for rare metabolic diseases, have advanced into clinical development.

While the intended therapeutic effect is mediated by translation of the encoded protein, the RNA and formulation can contribute to innate immune activation unless mitigated by nucleoside modification and purification.^3^^,^^22^ This necessitates chemical modifications (e.g., N1-methylpseudouridine), protective encapsulation (e.g., LNPs), and often high or repeated dosing to maintain therapeutic protein levels.^3^^,^^13^ Nevertheless, ongoing advances in formulation, stability, and targeted delivery are bringing mRNA-based protein replacement therapies closer to mainstream clinical use.^13^^,^^23^

### siRNA therapeutics

siRNAs are short, double-stranded RNA molecules, typically 21–23 nucleotides in length, that harness the endogenous RNAi pathway to achieve sequence-specific post-transcriptional gene silencing. Upon cellular entry, the guide (antisense) strand is incorporated into the RNA-induced silencing complex (RISC), where it directs cleavage and degradation of complementary mRNA targets.^24^

Several siRNA drugs have received regulatory approval, marking a milestone for RNA-based therapeutics. Patisiran (Onpattro) was the first FDA-approved siRNA drug in 2018, indicated for the treatment of hereditary transthyretin-mediated (hATTR) amyloidosis.^1^ It is delivered via LNPs and silences the expression of transthyretin in the liver. Since then, additional siRNA therapies have been approved: Givosiran (Givlaari) for acute hepatic porphyria,^25^ Lumasiran (Oxlumo) for primary hyperoxaluria type 1,^26^ Inclisiran (Leqvio) for lowering low-density lipoprotein (LDL) cholesterol in atherosclerotic cardiovascular disease,^9^ and Vutrisiran (Amvuttra), a subcutaneously delivered siRNA for hATTR amyloidosis.^10^ With the exception of patisiran, which is delivered via LNPs, these therapies leverage GalNAc conjugation for targeted delivery to hepatocytes via the asialoglycoprotein receptor.^14^

Beyond liver-directed applications, siRNAs are being actively explored in the treatment of oncology, viral infections, and ocular diseases. Preclinical studies support co-targeting of transforming growth factor β (TGF-β) and cyclooxygenase-2 (COX-2) in hepatocellular carcinoma.^27^ While peer-reviewed clinical data remain limited, earlier candidates such as TKM-080301, a PLK1-targeting siRNA formulated in LNPs, entered clinical trials for hepatocellular carcinoma but showed limited antitumor activity as monotherapy, highlighting early challenges in siRNA delivery and tumor targeting.^28^ In the context of viral infections, MIR 19, an inhaled siRNA therapeutic for COVID-19, has shown signals of clinical benefit in early-stage studies,^29^ while preclinical studies confirm the utility of LNP-formulated siRNAs for SARS-CoV-2 and other viruses.^30^ Ocular diseases are also being targeted, with agents like Tivanisiran under phase 3 evaluation for dry eye disease.^31^ Despite these advances, systemic and extrahepatic delivery of siRNAs remains a significant hurdle. Challenges include stability, rapid clearance, and tissue-specific uptake. To overcome these, researchers are developing advanced delivery strategies such as polymeric carriers, GalNAc conjugation, and nanoparticles to improve targeting and extend the half-life of siRNA drugs.^32^

### ASO therapeutics

ASOs are short, synthetic single-stranded nucleic acids designed to bind RNA via Watson-Crick base pairing to modulate gene expression.^33^ The first FDA-approved ASO therapy was fomivirsen, used in the late 1990s for cytomegalovirus retinitis.^34^ More recently, nusinersen (Spinraza), an ASO that modulates survival of motor neuron 2 (SMN2) gene splicing, has transformed treatment for spinal muscular atrophy, providing significant motor function gains and survival benefit in infants with the disease.^35^ Similarly, eteplirsen and related exon-skipping ASOs have been approved for subsets of Duchenne muscular dystrophy, demonstrating that ASO-mediated exon skipping can restore truncated but partially functional dystrophin protein.^36^

ASO therapies are also being developed for amyotrophic lateral sclerosis (ALS),^37^ Huntington’s disease,^38^ and other neurogenetic conditions, where central nervous system delivery (typically intrathecal) is feasible and therapeutic targets are well defined.^39^ Advances in formulation and conjugation technologies have enabled ASOs to access broader tissues, including the liver (e.g., for metabolic and cardiovascular indications) and kidney, but tissue-specific delivery remains an area of active research.^40^ AIC468 provides an emerging example of ASO-based antiviral therapy for kidney-associated BK polyomavirus (BKV) infection. AIC468 is currently in phase I clinical trials and has been designed to exploit the natural distribution of ASOs to proximal tubular epithelial cells (PTECs) to inhibit BKV replication in kidney transplant recipients.^41^^,^^42^

While ASOs have a favorable safety profile, their lack of intracellular amplification, need for repeated administration, and potential for off-target effects or immune stimulation require ongoing optimization.^43^ Nonetheless, ASOs represent a clinically validated and rapidly growing class of RNA therapeutics with several approved drugs and a broad development pipeline.

siRNAs and ASOs are both single-gene silencing platforms with strong clinical validation, but they differ in several ways that influence therapeutic choice. Mechanistically, siRNAs act exclusively in the cytoplasm via catalytic RISC-mediated mRNA cleavage,^24^^,^^44^ while ASOs can act in both the cytoplasm and nucleus, expanding their target space to include pre-mRNA splice sites, nuclear long non-coding RNAs, and intronic sequences inaccessible to RNAi.^8^^,^^45^ This gives ASOs a distinct advantage for splice-switching applications such as nusinersen^35^ and eteplirsen,^46^ where modification of pre-mRNA processing rather than mRNA degradation is the therapeutic goal. Conversely, siRNAs benefit from catalytic, multi-turnover silencing: a single RISC-loaded siRNA can cleave multiple mRNA molecules, enabling potent gene knockdown at low intracellular doses.^44^ For delivery, GalNAc-conjugated siRNAs have achieved hepatocyte targeting with subcutaneous dosing intervals of up to 6 months, matching or exceeding the durability of many ASO regimens.^9^^,^^10^ ASOs, however, retain advantages in tissue breadth: naked ASOs distribute to kidney, and intrathecal ASOs access the central nervous system without the need for nanoparticle formulation,^39^^,^^40^ enabling treatment of neurological diseases such as spinal muscular atrophy and ALS where siRNA delivery remains challenging.^35^^,^^37^ Both platforms share risks of off-target hybridization and, for some chemistries, immune stimulation,^32^^,^^43^ and both benefit from chemical modification strategies that have been extensively optimized over decades of development.^33^

### Aptamer and RNA decoy therapeutics

Aptamers are short, structured single-stranded RNAs that fold into specific three-dimensional conformations, enabling them to bind target molecules with high affinity and specificity.^47^ They are typically identified through the Systematic Evolution of Ligands by EXponential enrichment (SELEX) process.^48^^,^^49^ Functionally analogous to antibodies, aptamers can inhibit protein function, modulate signaling pathways, or serve as targeted delivery vehicles.

Despite their potential, the clinical translation of aptamers has faced challenges, including susceptibility to nuclease degradation and rapid renal clearance.^16^ To address these issues, various chemical modifications, such as 2′-fluoro, 2′-*O*-methyl substitutions, and polyethylene glycol (PEG) conjugation, have been employed to enhance stability and pharmacokinetics. Notably, pegaptanib (Macugen), an RNA aptamer targeting vascular endothelial growth factor (VEGF), was approved by the FDA in 2004 for the treatment of neovascular age-related macular degeneration.^17^

RNA decoys are synthetic oligonucleotides designed to mimic endogenous nucleic acid structures, thereby sequestering specific proteins or RNAs and modulating their activity. One prominent application involves transcription factor decoys (TFDs), which are double-stranded DNAs or RNAs containing consensus binding sites for transcription factors.^50^ By binding these factors, TFDs prevent them from interacting with their natural genomic targets, thus modulating gene expression. More recently, RNA decoys have been developed to target RNA-binding proteins involved in post-transcriptional regulation, including splicing factors such as RNA-binding fox-1 homolog 1/2 (RBFOX1/2) and serine and arginine-rich splicing factor 1 (SRSF1).^51^ These decoys can modulate alternative splicing or inhibit pathological RNA-protein interactions, expanding their therapeutic relevance beyond transcriptional control. Both transcription factor and splicing factor decoys have shown promise in preclinical models of cancer, cardiovascular disease, and neurological disorders.

While both aptamers and RNA decoys offer unique mechanisms for therapeutic intervention, their clinical development requires overcoming challenges related to stability, delivery, and specificity. Ongoing research and technological advancements continue to address these hurdles, aiming to harness the full potential of these nucleic acid-based therapeutics.

### miRNA therapeutics

Victor Ambros and Gary Ruvkun were awarded the 2024 Nobel Prize in Physiology or Medicine for their discovery of miRNAs (miRNAs), revealing a fundamental mechanism of post-transcriptional gene regulation in multicellular organisms. In 1993, Ambros and his team identified the first miRNA, lin-4, in the nematode Caenorhabditis elegans.^52^ Subsequently, Ruvkun’s research demonstrated that lin-4 regulates the lin-14 gene by binding to its mRNA, thereby inhibiting its translation into protein.^53^ This discovery revealed a novel layer of gene regulation, highlighting the crucial role of small non-coding RNAs in controlling gene expression.

miRNAs are endogenous, non-coding RNAs approximately 18–25 nucleotides in length that regulate gene expression post-transcriptionally by promoting mRNA degradation or inhibiting translation.^18^^,^^19^ Aberrant miRNA expression is implicated in a wide range of human diseases, including cancer, cardiovascular and metabolic disorders, and viral infections.^54^ Consequently, modulating miRNA activity has emerged as a therapeutic strategy, typically via miRNA mimics (to restore function) or antimiRs (to inhibit overactive or pathogenic miRNAs).^20^

One of the most clinically advanced miRNA-targeting therapeutics is miravirsen, an antimiR that binds and inhibits miR-122, a liver-specific miRNA essential for hepatitis C virus replication. Miravirsen showed dose-dependent, prolonged antiviral activity in a Phase 2a trial, establishing proof-of-concept for systemic antimiR therapy.^55^ A related locked nucleic acid (LNA)-modified antimiR, RG-101, also targeting miR-122, demonstrated promising results but was later halted due to safety concerns.^56^

In oncology, miRNA-based therapeutics have been investigated for tumor suppression or oncogene silencing. MRX34, a liposomal mimic of the tumor-suppressor miR-34a, was the first miRNA mimic to enter clinical trials for advanced solid tumors. While initial studies showed some signs of efficacy, the trial was terminated early due to immune-related serious adverse events.^57^ This highlighted the need for improved delivery and reduced immunostimulation in miRNA therapeutics.

miRNA targeting has also been explored in fibrotic, cardiovascular, and neurological diseases, where dysregulated miRNAs play a functional role in pathogenesis. For example, antimiR-21 has been studied in preclinical models of cardiac fibrosis, where inhibition improved cardiac function and reduced fibrotic remodeling.^58^ Similarly, antimiR-155 has demonstrated therapeutic potential in inflammatory and autoimmune diseases, including multiple sclerosis and amyotrophic lateral sclerosis, by modulating neuroinflammation and immune cell activation.^59^

Despite their broad mechanistic potential and regulatory scope, miRNA therapeutics face significant challenges in delivery, target specificity, and safety. miRNAs often regulate multiple genes, increasing the risk of off-target effects. Moreover, stable and tissue-specific delivery remains challenging, particularly for miRNA mimics, which resemble endogenous miRNAs and may be rapidly degraded or functionally diluted. Nevertheless, progress in miRNA therapeutics has been driven by refined chemical modifications (e.g., LNAs, 2′-*O*-methyl, phosphorothioates) and improved delivery vehicles (e.g., liposomes and nanoparticles), which have enhanced stability, potency, and tolerability, but have not yet fully resolved challenges related to tissue specificity and safety.^54^ While no miRNA-based drug has yet been approved as of June 2026, early clinical trials have demonstrated proof-of-mechanism, and despite several high-profile clinical discontinuations, newer agents are in development with improved chemistry and delivery systems aimed at better safety, tissue selectivity, and target specificity.

A related class of non-coding RNAs, long non-coding RNAs (lncRNAs), also modulate gene expression, but through diverse mechanisms such as chromatin remodeling, RNA sponging, and scaffolding of protein complexes.^60^ While lncRNA therapeutics remain largely in preclinical development, they represent a growing area of interest.

## Replicating RNA therapeutics

In contrast to non-replicating RNAs, replicating RNA therapeutics are capable of intracellular amplification, which can enhance potency, prolong activity, and modulate immune responses without necessarily requiring repeat administration.^61^^,^^62^ This category encompasses a range of platforms, from naturally derived defective viral genomes (DVGs) to saRNA constructs. These RNAs often encode, or co-opt in trans, viral replicase proteins to achieve autonomous RNA amplification within the cytoplasm, sometimes accompanied by expression of a therapeutic or immunogenic gene. While most replicating RNA platforms remain in preclinical or early clinical development, the recent approval of the saRNA COVID-19 vaccine ARCT-154 in Japan has demonstrated the clinical viability of this approach and may pave the way for broader therapeutic applications.^63^^,^^64^ A second self-amplifying mRNA product, GEMCOVAC-OM, an Omicron-specific booster developed by Gennova Biopharmaceuticals, received Emergency Use Authorization in India in 2023, further supporting the clinical viability of the platform; the earlier GEMCOVAC-19 was a conventional, non-amplifying mRNA vaccine.^65^ Table 2 summarizes key replicating RNA therapeutic platforms. It highlights their delivery strategies, distinguishing features, and representative clinical applications, illustrating the growing diversity and translational potential of these technologies.

**Table 2:** Replicating RNA therapeutics: Delivery strategies, mechanisms, and key considerations

RNA type	Common delivery platforms	Typical or explored routes	Notable features	Delivery challenges	Duration of action	References
saRNA	• LNPs • Polymers	intramuscular for vaccines intravenous, intratumoral, or mucosal routes under investigation	• intracellular amplification • strong immunogenicity • dose-sparing	• large size (∼10 kb) • innate immune activation • formulation complexity	moderate to durable (weeks; longer expression than mRNA from a single dose)	Casmil et al.,^61^ Oda et al.,^63^ Maine et al.,^66^ Frederickson and Herzog^67^
DI RNA	• VLPs • LNPs	Intranasal, intravenous, subcutaneous, or local delivery depending on virus/model and formulation	• requires helper virus • Dual mechanism via interference and innate immune activation	• helper virus dependence • specificity of delivery • standardization	transient to moderate (days to weeks; dependent on co-infecting helper virus)	Xiao et al.,^68^ Pitchai et al.,^69^ Rezelj et al.,^70^ Li et al.,^71^ Lin et al.,^72^ Lin et al.^73^
Viral replicon	• VLPs • electroporation	intramuscular, subcutaneous, intratumoral, or *ex vivo*/electroporation-based delivery depending on vector system	• Encodes replicase • produces payload RNA in infected cells without generating new virus	• requires structural gene support in trans • scalability for clinical use	moderate (days to weeks per administration)	Khromykh and Westaway,^74^ Lundstrom,^75^ Xiong et al.,^76^ Aliahmad et al.,^77^ Pushko and Tretyakova^78^
Synthetic RNA replicon/hybrid	• LNPs • plasmids	intramuscular, intravenous, intradermal, oral/bacterial, or *ex vivo* delivery depending on platform	• modular payload design • programmable regulatory elements • potential for self-regulation	• design complexity • stability • regulation of multi-RNA interactions	moderate (weeks; platform-dependent)	Zhao et al.,^79^ Wagner and Mutschler,^80^ Jawalagatti et al.,^81^ Yoshioka et al.,^82^ Wagner and Mutschler^83^

saRNA, self-amplifying RNA; LNPs, lipid nanoparticles; DI RNA, defective interfering RNA; VLPs, virus-like particles.

The replicating RNA platforms described in this section differ from one another along several key axes that have direct consequences for therapeutic design and clinical translation (Table 2). saRNAs, also referred to as replicon RNAs, encode their own replicase and replicate autonomously after delivery, while lacking the structural genes required for production of infectious progeny.^74^^,^^75^ This architecture makes saRNAs particularly amenable to LNP-based delivery and non-viral manufacturing. Replicon-based viral systems use the same fundamental replicon RNA architecture but employ viral particles to deliver the replicating RNA to target cells, with viral structural proteins supplied in trans during particle production. This enables efficient particle-mediated delivery followed by sustained intracellular RNA amplification, while the absence of structural genes from the replicon genome prevents the production of progeny particles and thereby restricts particle-mediated spread to a single round.^75^^,^^84^ Replication-competent viral vectors undergo full replication cycles including virion production and spread, generating the strongest innate immune activation and highest amplification but also the greatest regulatory burden and safety requirements.^75^^,^^84^ DI RNAs occupy a distinct position: they encode no replicase of their own and cannot replicate in the absence of a helper virus, yet their potent RIG-I and MDA5 activation gives them a strong innate immune signature disproportionate to their size.^6^^,^^85^ Across all platforms, payload capacity, innate immune stimulation, manufacturing scalability, and the need for helper functions vary substantially, and the optimal choice depends on the therapeutic indication, target tissue, and acceptable safety profile.^6^^,^^62^^,^^75^

### saRNA therapeutics

saRNAs, also termed replicon RNAs, are synthetic RNA constructs, most commonly derived from alphavirus genomes and less frequently from other positive-strand RNA viruses, that combine an RNA amplification module with a therapeutic payload.^86^^,^^87^ In vaccine applications, the payload is typically an antigen, and the construct is usually delivered using LNPs to protect the RNA and promote cellular uptake. Unlike conventional mRNAs, saRNAs are designed to amplify intracellularly after delivery, enabling higher and more sustained antigen expression from relatively small input doses of RNA. This dose-sparing potential allows saRNA vaccines to elicit potent immune responses at doses substantially lower than those used for conventional mRNA vaccines, which may reduce manufacturing costs and improve scalability. Moreover, because antigen expression can persist for longer than with non-replicating mRNA, saRNAs may induce more durable immune responses.^63^^,^^88^

The replicative properties that enhance potency are often assumed to increase innate immune activation and reactogenicity. In practice, much of the reactogenicity of *in vitro*-transcribed RNA products appears to be driven by the drug product and process-related impurities (particularly double-stranded RNA byproducts of *in vitro* transcription), together with the LNP carrier, rather than by active intracellular replication; consistent with this, acute reactogenicity typically resolves within a few days whereas saRNA replication continues beyond that window.^89^^,^^90^ Rigorous safety comparison between replicating and non-replicating RNA therefore requires head-to-head studies in which formulation, dose, and manufacturing are matched.^89^^,^^90^ As such, saRNA platforms must balance potency with tolerability through careful optimization of RNA design, replicase engineering, and formulation, most commonly using LNPs.^61^^,^^88^^,^^91^

The examples in Table 3 illustrate the growing momentum behind saRNA as a versatile and scalable vaccine platform, with applications extending beyond SARS-CoV-2. Candidate vaccines targeting influenza virus, Zika virus (ZIKV), human immunodeficiency virus (HIV), rabies virus, and respiratory syncytial virus (RSV) are under investigation,^66^^,^^95^ and the technology is increasingly being explored in oncology for tumor-specific antigen delivery.^96^

**Table 3:** Examples of saRNA vaccine platforms in development

Vaccine/Platform	Target indications	Technology	Clinical stage	Reference
ARCT-154 (Arcturus Therapeutics)	COVID-19	saRNA	approved in Japan (2023)	Oda et al.,^63^ Ho et al.^64^
GRT-R910 (Gritstone bio)	COVID-19 (booster, broad variant protection)	saRNA encoding spike protein and replicase	phase 1 clinical trials	Palmer et al.^92^
Imperial College London saRNA platform	COVID-19 (early clinical trials)	saRNA COVID-19 vaccine (LNP-nCoVsaRNA)	first-in-human trials (2020)	McKay et al.^93^
RBI-4000 (Replicate Biosciences)	Rabies	srRNA	phase 1 clinical trials	Maine et al.,^66^ Maine et al.^94^
GEMCOVAC-OM (Gennova Biopharmaceuticals)^a^	COVID-19 (Omicron booster)	saRNA (LNP)	emergency use authorization, India (2023)	Saraf et al.^65^

a GEMCOVAC-19, an earlier non-amplifying mRNA vaccine from the same developer, is not included in this table as it does not use self-amplifying RNA technology. LNP, lipid nanoparticle; srRNA, synthetic replicon RNA.

To fully realize this potential, ongoing research is focused on improving saRNA design, delivery, immunogenicity, and tolerability. Key areas include engineering less immunostimulatory replicase systems, developing delivery vectors for muscle and mucosal tissues, combining saRNA with adjuvants or self-assembling nanoparticle antigens, and evaluating durability, immune memory, and dose-sparing performance across diverse populations.^87^ Although complete nucleoside substitution was long considered incompatible with saRNA replication, McGee et al. demonstrated that 100% substitution with 5-methylcytidine (m5C) preserves replicase function, suppresses the interferon response, and enhances protein expression up to 68-fold over N1-methylpseudouridine (N1mΨ) mRNA *in vitro*, with 75% survival against lethal SARS-CoV-2 challenge at a 10 ng vaccine dose in mice.^97^

### Replicon-based viral systems

Replicon-based viral systems are engineered RNA virus-derived systems that retain the ability to replicate their genomes intracellularly but are modified to prevent the production of infectious progeny. Early pioneering work in this field during the late 1980s and 1990s by researchers including Charles Rice, Kenneth Lundstrom, and Robert Johnston established the foundations for alphavirus- and flavivirus-derived replicon systems, particularly using Sindbis virus, Semliki Forest virus (SFV), Venezuelan equine encephalitis virus (VEEV), and Kunjin virus as self-replicating RNA delivery platforms.^74^^,^^76^ In these systems, the viral non-structural genes encoding the RNA-dependent RNA polymerase (RdRp) complex are retained in the replicon RNA, while the structural genes essential for particle assembly are deleted from the replicon genome and supplied in trans during particle production, enabling robust intracellular RNA amplification following delivery without generation of replication-competent virus.

The self-amplifying nature of replicon-based viral systems allows sustained and high-level expression of encoded antigens or therapeutic proteins, making them attractive for vaccine development and cancer immunotherapy. Alphavirus-derived replicon particles have been explored extensively in preclinical models and early-stage clinical studies, where they induce strong innate and adaptive immune responses and promote antigen-specific T cell activation.^62^^,^^77^ More recently, replicon-based viral systems derived from other RNA viruses, including rhabdoviruses (e.g., VSV) and paramyxoviruses (e.g., measles virus), have expanded the diversity of viral backbones available for replicon development.^62^

In cancer immunotherapy, alphavirus-derived replicon particles based on SFV and VEEV have been engineered to express tumor-associated antigens or immunostimulatory molecules, resulting in robust activation of cellular and humoral immune responses.

Preclinical models demonstrate that these systems can mediate tumor regression and prolonged survival in mice bearing melanoma, breast, brain, and cervical tumors, attributed to both intracellular cytopathic effects and the induction of antigen-specific T cell responses.^75^^,^^77^

Despite their promise, challenges remain in the clinical application of replicon-based viral systems, including vector- or particle-associated innate immune activation that can limit transgene expression, pre-existing immunity to viral components, and scalability of manufacturing.^67^^,^^75^ Ongoing research aims to address these limitations through improved replicon design, optimized delivery strategies, and a deeper understanding of host-vector interactions.

### Replication-competent viral vectors

Replication-competent viral vectors represent a distinct class of self-amplifying nucleic acid therapeutics in which the viral genome retains the capacity for full replication and production of infectious progeny, albeit in an attenuated or genetically modified form. Unlike saRNA constructs or viral replicon particles, these vectors undergo complete viral replication cycles within host cells, including genome amplification, expression of structural proteins, virion assembly, and cell-to-cell spread.^75^^,^^84^ Attenuation strategies, such as deletion or replacement of virulence factors or substitution of viral glycoproteins, are employed to balance immunogenicity with acceptable safety profiles.^98^

Several RNA viruses have been adapted as replication-competent vectors, including vesicular stomatitis virus (VSV) and measles virus. These platforms exploit the inherent immunogenicity and amplification capacity of live viruses to drive potent innate and adaptive immune responses, particularly in vaccine and oncolytic applications.^84^ Because replication-competent vectors generate progeny virions, they differ fundamentally from viral replicon particles, which amplify RNA intracellularly but are incapable of producing infectious particles, and from saRNA systems, which encode viral replicase but lack all structural components required for virion formation.^74^

A prominent clinical example of a replication-competent viral vector is the recombinant vesicular stomatitis virus-based Ebola vaccine rVSV-ZEBOV (Ervebo), in which the VSV glycoprotein is replaced with the Ebola virus glycoprotein. This vector retains full replication competence while exhibiting attenuated pathogenicity in humans.^99^ In a landmark ring vaccination trial conducted during the West African Ebola outbreak, rVSV-ZEBOV demonstrated high efficacy in preventing Ebola virus disease, providing proof-of-concept that replication-competent viral vectors can be deployed safely and effectively in outbreak settings.^98^ The success of rVSV-ZEBOV underscores the translational potential of this platform but also highlights regulatory and biosafety considerations unique to replication-competent systems.

Replication-competent viral vectors have also been explored extensively in cancer immunotherapy, where their ability to spread within tissues and induce local inflammation can enhance anti-tumor immunity. Oncolytic viruses, including modified VSV and measles virus strains, leverage selective replication in tumor cells to mediate direct oncolysis while simultaneously stimulating systemic immune responses.^84^ However, the capacity for uncontrolled replication, pre-existing immunity, vector shedding, and off-target tissue infection necessitates stringent attenuation strategies and careful clinical monitoring.

From a therapeutic design perspective, replication-competent viral vectors occupy a distinct position along the RNA replication spectrum. Their ability to amplify both RNA and viral particles confers unmatched immunostimulatory potency but limits their suitability for applications requiring precise dosing, repeated administration, or minimal systemic inflammation. Consequently, while replication-competent viral vectors have achieved clinical use in select infectious disease and oncology contexts, their use contrasts sharply with replicon RNAs and DI RNAs, which seek to decouple intracellular RNA amplification from viral propagation.^62^^,^^75^ Understanding these distinctions is critical for rational selection of self-amplifying platforms based on therapeutic intent, safety requirements, and translational feasibility.

### Synthetic and hybrid replicating RNA platforms

Beyond conventional replicon architectures, synthetic and hybrid RNA platforms have emerged to expand delivery control and functional diversity of replicating RNAs. This category is defined by engineering at the level of the replicon RNA architecture itself, not by delivery format: an alphavirus saRNA delivered in LNPs remains a saRNA (covered in the preceding section) and is not a synthetic or hybrid platform merely by virtue of its LNP formulation. Rather, synthetic and hybrid platforms are distinguished by novel replicase engineering, modular payload design, non-alphaviral amplification elements, or fusion of replicating and non-replicating functional modules in ways that create fundamentally new RNA architectures. Advances in synthetic biology have facilitated the design of programmable RNA replicons and hybrid systems that merge self-amplifying elements with regulatory modules, RNA sensors, or therapeutic payloads.^79^^,^^100^ These innovations aim to harness the benefits of intracellular RNA amplification while enhancing precision and safety.^87^ For example, alphavirus replicon genomes have been engineered as modular expression systems capable of supporting high-level expression of heterologous payloads, providing a foundation for designs that combine replicating RNA backbones with therapeutic or regulatory RNA elements.^101^^,^^102^ In addition, bacteriophage-derived elements have been adapted as engineered RNA amplification systems capable of sustained intracellular protein production, with expression properties tunable through RNA structure and sequence design.^80^ Together, these systems exemplify modular replicating RNA platforms capable of co-expressing therapeutic RNAs and proteins while enabling tighter control over RNA amplification and immunogenicity than naturally derived viral replicon RNA architectures.^79^^,^^80^^,^^100^

The development of synthetic replicon vaccines has prompted a diverse set of delivery strategies aimed at overcoming challenges of stability, transfection efficiency, and immune evasion. Among non-viral platforms, lipid-based nanoemulsions, dendrimers, and polymeric micelles are widely explored for their ability to protect RNA from nuclease degradation and enable cytosolic delivery. Certain lipid-based nanoemulsion systems have been shown to form stable electrostatic complexes with replicon and to retain functional activity during extended storage at ambient temperatures, supporting deployment in resource-limited settings.^103^ Related lipid-inorganic composite formulations, including LION-based platforms, similarly promote RNA complexation and stability relative to conventional LNPs, although long-term ambient storage properties vary by formulation.^91^ Modified dendrimer-based nanoparticles (MDNPs) have demonstrated large payload capacity and durable protection, with recent formulations generating long-term immunity *in vivo* against pathogens such as Toxoplasma gondii.^100^ Ionizable micelles containing phenylalanine moieties induce pH-sensitive endosomal escape, enhancing cytoplasmic release and transgene expression.^104^ These examples illustrate representative strategies rather than an exhaustive catalog of delivery systems, highlighting the diversity of approaches being applied to synthetic replicon RNA platforms.

Coatsome-based polyplexes and cationic nanolipoprotein particles are also being developed to optimize replicon complexation and gene expression, with tunable physicochemical properties tailored for specific cell types.^105^ Other polymer-based vehicles, including PEI polyplexes, have been explored for targeting dendritic cells and enhancing immune priming.^106^ Parallel to RNA formulations, DNA-launched replicons represent an alternative platform in which eukaryotic promoters such as cytomegalovirus (CMV) drive intracellular transcription of a replicon from plasmid DNA following nuclear delivery. One representative approach used an SFV-derived DNA-launched RNA replicon delivered by attenuated *Salmonella* through bactofection, enabling *in vivo* delivery and multicistronic SARS-CoV-2 antigen expression.^81^ Although these DNA-launched systems retain the production and stability advantages of plasmid DNA, their dependence on nuclear delivery distinguishes them from direct RNA replicon delivery.

Another hybrid innovation is the insect-specific virus (ISV) vector system, including insect-specific alphaviruses (ISAVs) and insect-specific flaviviruses (ISFs), which replicate efficiently in insect cells but are replication-deficient in mammalian cells. This restriction arises from host-specific barriers at the levels of entry, translation, RNA replication, or virion assembly.^107^ Their inability to propagate in mammalian cells makes ISVs attractive platforms for producing viral RNAs and virus-like particles (VLPs) that retain immunogenicity without the risks associated with *in vivo* replication. Chimeric ISVs have been engineered by replacing their structural genes with those from vertebrate-infecting arboviruses. One prominent example is the use of Binjari virus, an ISF, as a backbone to express Japanese encephalitis virus prM and E proteins, resulting in VLPs that can enter mammalian cells and elicit protective immune responses while remaining replication-deficient *in vivo*.^108^ These chimeric viruses replicate efficiently in mosquito cell lines but do not replicate in vertebrate cells, making them promising candidates for safe and immunogenic vaccine platforms. Similar Binjari virus-based chimeric vaccine approaches have also been demonstrated for West Nile Virus/Kunjin virus structural proteins.^109^

Collectively, these synthetic and hybrid designs aim to retain the potency of replicating systems while enhancing specificity, safety, and regulatory control. Hybrid replicons offer an attractive alternative where traditional non-replicating RNA would be too transient, or where natural viral replicon RNA may provoke excessive immune responses or impose payload constraints. Compared with replication-competent viral vectors, synthetic and hybrid replicon platforms may offer more tractable regulatory pathways due to the absence of productive infection, although their novelty presents distinct evaluation challenges. Vector design must therefore balance amplification efficiency with precise control of expression, immunogenicity, and off-target effects.

### DI RNAs as emerging therapeutic platforms

DI RNAs, also known as DVGs, are truncated RNA species that arise naturally during the replication of many RNA viruses.^6^^,^^7^^,^^110^ These molecules retain essential *cis*-acting sequences required for replication and packaging but harbor large internal deletions that eliminate their ability to encode functional viral proteins.^111^ As such, DI RNAs depend on a co-infecting wild-type (WT) virus to provide the missing replication machinery in trans.^112^ Once present, they are believed to outcompete full-length genomes for access to viral replicase, thereby suppressing WT virus replication. In addition, their RNA structure and replication make them potent activators of cytoplasmic RNA sensors such as RIG-I and MDA5, leading to robust interferon responses and antiviral gene expression.^85^ Although no DI RNA-based therapeutic has yet been approved, accumulating preclinical evidence supports their potential as a new class of antiviral intervention.

DVGs have been described for a variety of RNA viruses, including members of the *Flaviviridae*, *Picornaviridae*, *Paramyxoviridae*, *Orthomyxoviridae*, *Rhabdoviridae*, and *Coronaviridae* families.^6^ For example, engineered therapeutic interfering particles (TIPs), although distinct from directly administered DI RNA therapeutics, provide important proof-of-concept that DVG-derived systems can be harnessed therapeutically. Enteroviral TIPs derived from poliovirus DVGs have been shown to reduce viral replication and block transmission *in vivo*.^68^ In HIV-1, engineered TIPs derived from HIV-1 DI RNAs demonstrated strong antiviral activity in a chimeric SHIV-infected macaque model, where they significantly reduced plasma viral loads.^69^

ZIKV naturally generates DI RNAs during infection, particularly under high multiplicity of infection (MOI) conditions. These DI RNAs were identified through deep sequencing and functional analysis, and they interfere with virus replication and modulate host responses without requiring rational design.^70^

Another example is DI290, a non-coding DI RNA naturally derived from dengue virus (DENV) that retains replication-competent untranslated regions (UTRs).^71^ DI290 does not express viral proteins yet exhibits potent antiviral activity against DENV and other RNA viruses including ZIKV, yellow fever virus, RSV, and SARS-CoV-2.^72^ Its mechanism involves robust RIG-I-mediated interferon stimulation, and it has been validated in both cell culture and mouse models.^73^

Given their high specificity, inability to propagate independently, and strong immunostimulatory profiles, DI RNAs represent promising therapeutic modalities for outbreak settings, particularly where rapid, self-limiting antiviral activity is desirable. The expanding catalog of natural and synthetic DI RNAs offers exciting opportunities to develop safe, self-limiting antivirals tailored to specific pathogens.

### Replicating RNAs beyond vaccines and immunotherapy

Although the clinical development of replicating RNA platforms has been driven largely by vaccine applications, their capacity for intracellular amplification and sustained payload expression makes them attractive for a broader range of therapeutic contexts. Several areas are emerging as particularly promising.

In protein replacement therapy, saRNA has been explored as an alternative to conventional mRNA for disorders requiring sustained protein expression, including controllable erythropoietin delivery^113^ and coagulation factor expression in hemophilia.^61^^,^^114^ The amplification advantage means that therapeutic protein levels can potentially be maintained from a lower and less frequent dose than would be required with conventional mRNA, reducing the manufacturing burden and improving patient convenience.^61^^,^^114^ However, the intrinsic immunogenicity of saRNA must be carefully managed in non-immunological therapeutic settings, where interferon induction is undesirable rather than adjuvant.

In oncology, replicating RNA platforms offer several theoretical advantages over non-replicating formats. Replicon-based viral systems have been used to deliver tumor-associated antigens, cytokines such as interleukin-12, and immune checkpoint modulators directly to the tumor microenvironment, where localized amplification can sustain therapeutic concentrations.^75^^,^^77^ DI RNAs and TIPs represent a distinct oncology-adjacent opportunity: their potent RIG-I and MDA5 activation has the potential to reprogram the immunosuppressive tumor microenvironment toward an interferon-high, pro-inflammatory state, which could in principle sensitize tumors to checkpoint blockade.^6^^,^^115^

In regenerative medicine, synthetic alphavirus replicon RNAs encoding reprogramming transcription factors such as OCT4, SOX2, KLF4, and c-MYC have been used to generate induced pluripotent stem cells (iPSCs) without genomic integration, addressing a key safety limitation of viral vector-based approaches.^82^ The self-limiting nature of RNA replication is here an asset: expression is sustained long enough to achieve reprogramming but does not persist indefinitely.

Collectively, these applications illustrate that replicating RNA platforms are not inherently vaccine-specific technologies. Rather, intracellular amplification is a general pharmacological mechanism whose therapeutic value depends on matching the duration and intensity of expression to the biological requirement of the indication. Realizing this broader potential will require platform-specific solutions to immunogenicity management, particularly the development of fully modified saRNAs^97^ and inducible or self-limiting replicase systems that afford greater control over expression kinetics.^79^^,^^100^

## Mechanisms of action

The biological activity of RNA therapeutics is defined by how they interact with host cells following delivery. These interactions differ fundamentally between non-replicating RNAs, which exert their effect through single-round functional engagement with the cellular machinery and replicating RNAs, which undergo intracellular amplification and may persist longer or exert multi-modal effects. Understanding these mechanisms is essential for predicting therapeutic kinetics, immune responses, and safety profiles. Figures 1 and 2 illustrate the mechanisms of action of RNA therapeutic platforms, highlighting the molecular basis of non-replicating and replicating RNA-based therapies.

**Figure 1:**
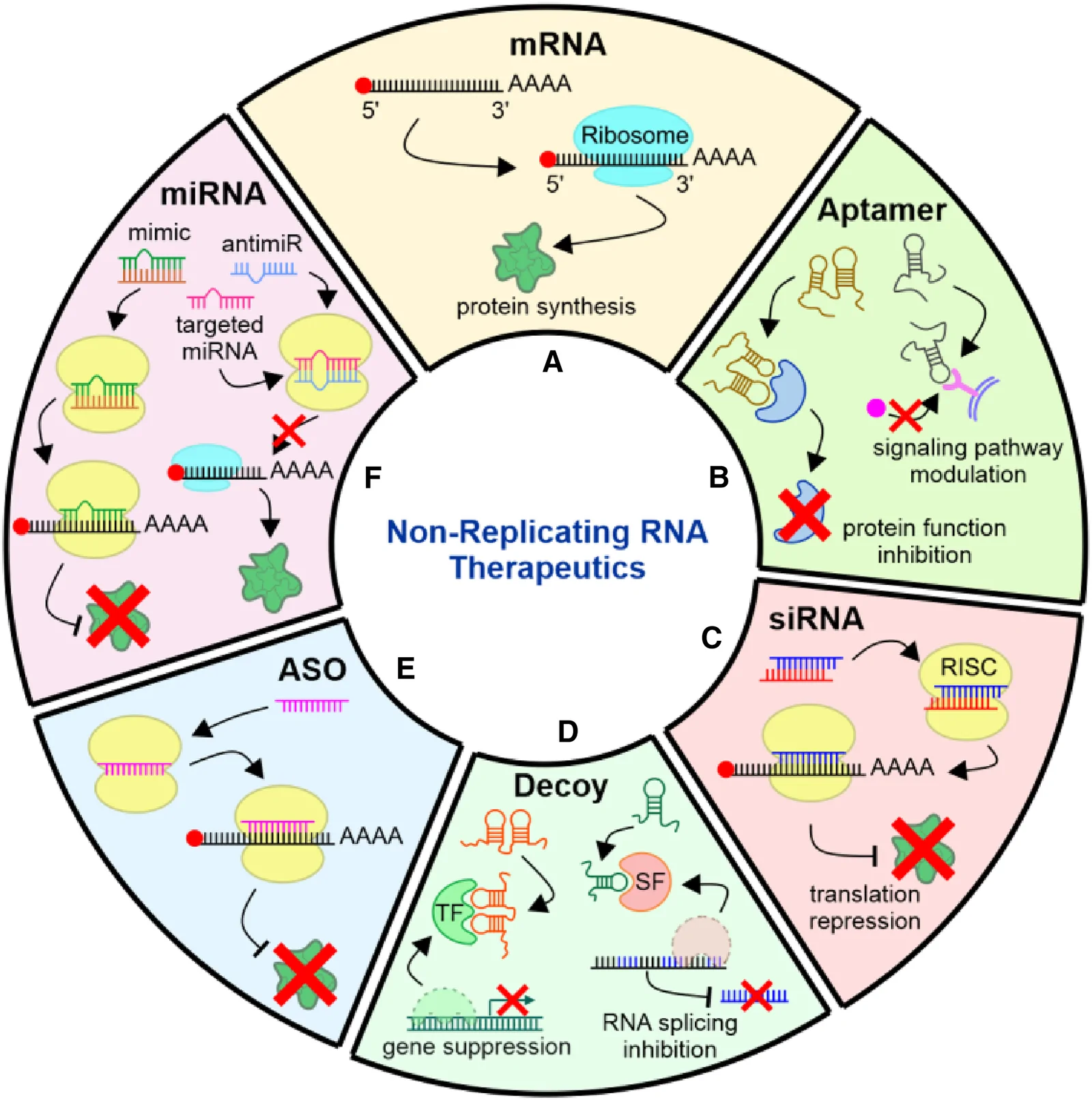
Mechanisms of non-replicating RNA therapeutics Schematic representation of six major non-replicating RNA therapeutic modalities. (A) mRNA therapeutics deliver coding transcripts that are translated by ribosomes to produce therapeutic proteins. (B) RNA aptamers fold into defined structures that bind target proteins, thereby inhibiting protein function or modulating signaling pathways. (C) siRNAs are incorporated into the RISC, where they direct sequence-specific target mRNA degradation and/or translational repression. (D) RNA decoys sequester TFs, SFs, or other regulatory proteins to suppress gene expression or alter RNA processing. (E) ASOs bind complementary RNA targets to inhibit translation, promote RNA degradation, or modulate splicing. (F) miRNA therapeutics include miRNA mimics and antimiRs. miRNA mimics associate with RISC to regulate target mRNAs through translational repression or mRNA degradation, whereas antimiRs bind endogenous miRNAs and prevent repression of their target transcripts.

**Figure 2:**
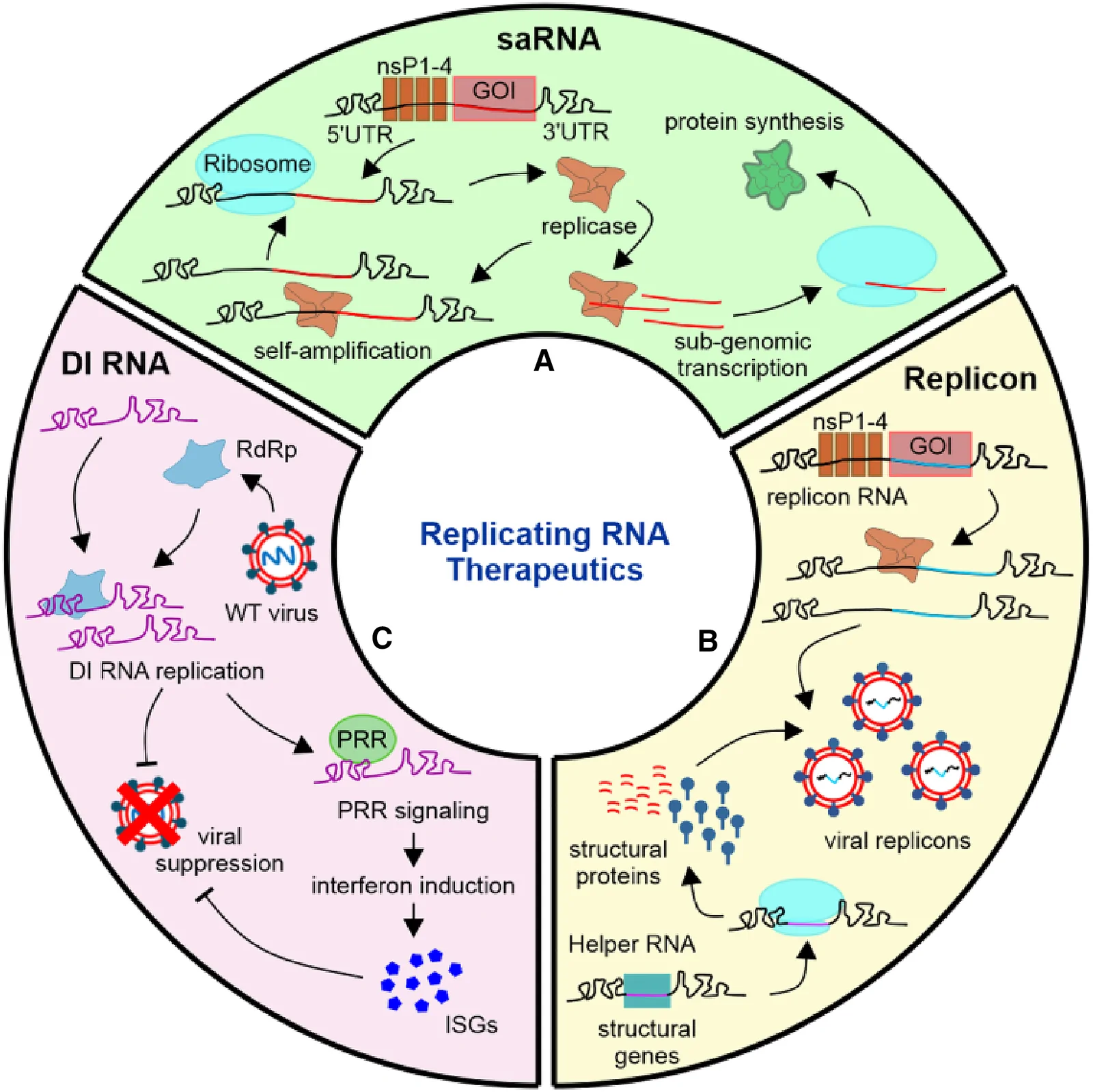
Mechanisms of replicating RNA therapeutics Schematic representation of three major replicating RNA therapeutic platforms. (A) saRNAs encode viral non-structural proteins, typically alphavirus nsP1-4, together with a gene of interest (GOI). Following cytoplasmic delivery, the replicase is translated and drives RNA self-amplification and subgenomic transcription, resulting in enhanced payload expression. (B) Replicon RNAs retain viral replicase genes and *cis*-acting replication elements but lack structural genes required for autonomous virion production. Structural proteins can be supplied in trans by helper RNAs or producer systems, enabling formation of viral replicon particles that deliver replicon RNA to target cells but do not undergo further propagation. (C) DI RNAs are truncated viral RNAs that retain replication elements but depend on a co-infecting wild-type (WT) virus to provide RdRp and other viral functions. DI RNAs can suppress WT virus replication through replicative interference and can activate PRRs, leading to interferon induction and expression of ISGs.

### Non-replicating RNAs

Non-replicating RNA therapeutics function without intracellular amplification and typically require precise design and delivery to achieve sufficient intracellular concentrations. Each subclass acts via a distinct mechanism.

#### Messenger RNA

mRNA therapies deliver capped and polyadenylated transcripts into the cytoplasm, where host ribosomes translate them into therapeutic proteins such as enzymes, antigens, or cytokines.^23^ Protein expression begins rapidly but is transient, limited by RNA degradation pathways and cellular turnover, often necessitating repeated dosing to maintain therapeutic levels. To enhance translation efficiency and stability, mRNAs are engineered with optimized 5′ cap structures, UTRs, and chemically modified nucleotides (e.g., pseudouridine).^3^ These modifications also reduce recognition by innate immune sensors, which can otherwise hinder translation through protein kinase R (PKR) activation or trigger inflammation.

#### siRNAs and ASOs

siRNAs are processed from longer double-stranded RNA precursors by Dicer, yielding ∼21–23 nucleotide duplexes. One strand is loaded into the Argonaute 2 (AGO2) protein within the RISC, facilitated by chaperones and guide strand asymmetry recognition. The AGO2-siRNA complex then binds fully complementary mRNA targets, catalyzing precise endonucleolytic cleavage via its PIWI domain, akin to RNase H-like activity. Structural studies have elucidated how AGO2 coordinates siRNA positioning and target cleavage, demonstrating an ordered sequence of guide strand loading, duplex unwinding, target binding, and catalysis.^44^

ASOs, in contrast, are typically single-stranded DNA-RNA chimeras designed to hybridize to target RNA and modulate their function. They operate through several mechanisms, including steric blocking of translation, modulation of splicing, or recruitment of RNase H1 for mRNA degradation.^15^^,^^45^ Gapmer ASOs feature a central DNA region flanked by modified nucleotides (e.g., 2′-*O*-methoxyethyl or LNAs) that improve nuclease resistance and enhance binding affinity. When these ASOs bind to their RNA, they recruit cellular RNase H1, which cleaves the RNA strand of the DNA-RNA hybrid, leading to mRNA degradation. Alternatively, fully modified ASOs (steric blockers), which lack a central DNA gap, can interfere with splicing or translation by sterically hindering ribosomal or spliceosomal components.^8^

#### Aptamers and RNA decoys

Aptamers and RNA decoys exert their therapeutic effects through molecular mimicry or high-affinity binding to cellular targets. Aptamers are short, structured RNAs that fold into defined three-dimensional shapes, enabling them to recognize and bind proteins, receptors, or small molecules with nanomolar-to-picomolar affinity, similar to monoclonal antibodies. They are typically identified through iterative *in vitro* selection techniques such as systematic evolution of ligands by exponential enrichment (SELEX), which have been enhanced by *in vivo* or capillary electrophoresis-based approaches to improve binding specificity and therapeutic utility.^49^ Chemical modifications such as 2′-fluoro and 2′-*O*-methyl substitutions further enhance aptamer stability and resistance to nuclease degradation. Upon target engagement, aptamers can inhibit ligand-receptor interactions, block enzyme activity, or allosterically modulate signaling pathways, allowing for reversible and tunable control of biological processes. A classic example from the broader aptamer field is the thrombin-binding aptamer, a single-stranded DNA aptamer that binds thrombin with high affinity and inhibits its protease activity, demonstrating how aptamers can directly modulate protein function.^116^ RNA decoys, by contrast, are designed to sequester regulatory proteins or RNAs by mimicking endogenous binding motifs. TFD oligonucleotides, often double-stranded DNA or RNA oligonucleotides, bind factors such as E2F transcription factor, nuclear factor-κB (NF-κB), or signal transducer and activator of transcription 3 (STAT3), preventing them from accessing genomic targets. This has produced therapeutic effects in cardiovascular injury and cancer models.^50^ Similarly, miRNA decoys effectively inhibit oncogenic miRNAs by providing multiple binding sites, reactivating cellular pathways in preclinical models.^117^

#### miRNAs

miRNAs are small non-coding RNAs that regulate gene expression post-transcriptionally. Their biogenesis begins with transcription from DNA into primary miRNAs (pri-miRNAs). In the nucleus, pri-miRNAs are processed into precursor miRNAs (pre-miRNAs) by a microprocessor complex composed of the RNA-binding protein DiGeorge Syndrome Critical Region 8 (DGCR8) and the RNase III enzyme Drosha.^118^ Once pre-miRNAs are generated, they are exported to the cytoplasm via the exportin-5/Ran-GTP complex.^119^ In the cytoplasm, pre-miRNAs are further cleaved by the Dicer into a mature miRNA duplex. One strand of this duplex, the guide strand, is incorporated into an Argonaute protein to form the RISC.^19^ The mature miRNA within RISC directs the complex to partially complementary target sequences, most commonly within the 3′ UTR of mRNAs. This interaction leads to gene silencing through several mechanisms: (1) inhibition of translation initiation by interfering with initiation factors or ribosome assembly; (2) mRNA destabilization through deadenylation and removal of the poly(A) tail, followed by decapping and exonucleolytic decay; and (3) direct mRNA cleavage if there is sufficient complementarity between miRNA and its target.^120^ Beyond the 3′ UTR, miRNAs can also bind to other regions of mRNAs, such as the 5′ UTR or coding sequences, and under certain conditions, may even enhance translation or participate in transcriptional regulation. In addition to their intracellular functions, miRNAs can be packaged into exosomes and secreted, allowing them to mediate intercellular communication.^121^ As therapeutic agents, antimiRs (also termed anti-miRs or antagomirs) act by the converse mechanism: they are chemically modified, single-stranded oligonucleotides that hybridize through Watson-Crick base pairing to the mature guide miRNA, sterically blocking and sequestering it from the RISC and thereby de-repressing that miRNA’s target mRNAs.^55^^,^^122^ Because the mature miRNA exerts its silencing activity predominantly within the cytoplasmic RISC, antimiR activity occurs mainly in the cytoplasm, although chemistries such as LNAs can also engage nuclear miRNA pools.^122^ In contrast, miRNA mimics are double-stranded RNAs that restore the function of a deficient miRNA by loading into the RISC in the manner of an endogenous duplex.^19^

### Replicating RNAs

Replicating RNA therapeutics differ from non-replicating RNAs by harnessing intracellular amplification via virus-derived replicase proteins. Once inside the cell, these RNAs (where encoded) are translated to produce a replicase complex, which drives the synthesis of complementary RNA strands and amplifies the intracellular abundance of the therapeutic or immunogenic payload. This amplification enhances protein output, prolongs expression, and often triggers innate immune responses through the generation of double-stranded RNA intermediates and 5′-triphosphate ends.

#### saRNAs

Mechanistically, saRNAs differ from conventional mRNAs because they encode a viral replicase, most commonly alphavirus nsP1-4 proteins, that amplifies the RNA after cytoplasmic delivery.^86^^,^^87^ Following translation of the replicase, the RdRp complex initiates synthesis of negative-strand RNA intermediates, which subsequently serve as templates for the production of positive-strand progeny RNAs. This process generates both full-length replicase-encoding RNAs and subgenomic transcripts that drive high-level payload expression, resulting in prolonged and abundant antigen or therapeutic protein production.^87^

saRNA replication also produces double-stranded RNA intermediates and other immunostimulatory RNA species such as 5′-triphosphate RNAs.^62^ These are recognized by a variety of host pattern recognition receptors (PRRs), including RIG-I^123^ and MDA5,^124^ which trigger the activation of interferon pathways and upregulation of interferon-stimulated genes (ISGs). Additionally, endosomal toll-like receptors (TLRs), particularly TLR3 (which detects double-stranded RNA) and TLR7/8 (which sense single-stranded RNA), can be engaged depending on the delivery route and subcellular localization of the saRNA.^125^ The resulting innate immune activation promotes antigen presentation and adaptive immune priming, enhancing the immunogenicity of replicating RNA vaccines and immunotherapies.^88^ However, excessive or systemic activation of these pathways can also induce inflammation, reduce protein translation, or cause dose-limiting toxicity, necessitating careful control over vector design, delivery, and dosing strategies.^91^ It should be noted that, while recognition of double-stranded RNA by RIG-I and MDA5 is firmly established experimentally, the extent to which replication-derived dsRNA intermediates, as distinct from process-related dsRNA impurities and the delivery vehicle, drive the reactogenicity of replicating RNA drug products *in vivo* remains largely inferred rather than directly demonstrated, and should be regarded as a working hypothesis pending matched, head-to-head data.^89^^,^^90^

#### Replicon-based viral systems

In replicon-based viral systems, the principal distinction from directly delivered saRNA lies in the mode of RNA delivery rather than in the fundamental mechanism of intracellular RNA amplification. Replicon genome is packaged into viral replicon particles using viral structural proteins supplied in trans during particle production, typically including capsid and envelope glycoproteins. This can be achieved by co-transfecting with helper RNAs or using stable producer cell lines that express the required structural proteins. The resulting viral replicon particles deliver the replicon RNA to target cells, where it undergoes intracellular amplification and drives therapeutic protein expression. Because the structural genes are absent from the replicon genome, newly infected cells cannot produce progeny particles. Viral replicon particles therefore enable efficient delivery and sustained intracellular RNA amplification while restricting the system to a single round of particle-mediated transfer, providing biological containment relative to replication-competent viral vectors.

#### DI RNAs

DI RNAs retain *cis*-acting replication elements, such as the 5′ and 3′ UTRs, replication promoters, and packaging signals, but contain internal deletions that eliminate most or all viral protein-coding capacity.^6^ Lacking these essential genes, DI RNAs cannot replicate independently and require a co-infecting WT helper virus to supply the missing *trans*-acting replication machinery.^111^^,^^112^^,^^126^ Once delivered into the cytoplasm alongside the helper virus, DI RNAs are efficiently replicated by the viral RdRp. Their shortened genomes are preferentially amplified, leading to higher intracellular abundance and allowing them to outcompete full-length viral genomes for access to the replication complex. This replicative interference reduces WT virus replication.^111^ The WT helper virus also provides the structural proteins required to encapsulate DI RNAs into transmissible VLPs, allowing DI RNAs to be replicated and packaged in the presence of a co-infecting helper virus and efficiently interferes with WT viral replication.^110^^,^^127^^,^^128^

Concurrently, DI RNAs activate host innate immune responses through the formation of double-stranded RNA intermediates and exposed 5′-triphosphate ends, which are recognized by cytoplasmic PRRs.^115^^,^^129^ This triggers robust interferon production and upregulation of ISGs, establishing an antiviral state in infected and neighboring cells.

Historically, most DI RNAs are non-coding, though some retain partial open reading frames and can express truncated proteins. For example, a ZIKV-derived DVG studied by Rezelj et al. was shown to produce a defective viral protein, indicating that the coding capacity of DI RNAs may influence their immunogenicity and therapeutic effects.^70^

DI RNAs have been identified across numerous RNA viruses, including DENV,^71^ influenza A virus,^110^ measles virus,^130^ polio virus,^131^ HIV-1,^69^ and SARS-CoV-2,^132^ and can be either naturally occurring or synthetically designed.^69^^,^^132^ DI290, a DI RNA derived from DENV that retains replication-competent UTRs but lacks protein-coding sequences, was developed and characterized as a potent RIG-I-mediated interferon inducer with antiviral efficacy *in vitro* and in mouse models against multiple RNA viruses.^72^^,^^133^

Thus, DI RNAs exert their therapeutic effects through a dual mechanism: (1) competitive inhibition of WT virus replication and (2) stimulation of innate antiviral immunity. Their inability to propagate independently and their replication-defective nature and dependence on helper virus functions confer a favorable intrinsic safety profile, making them attractive candidates for antiviral therapy,^7^ particularly in outbreak scenarios or immunocompromised populations.

## Safety and immunogenicity considerations

The safety and immunogenicity profiles of RNA therapeutics are shaped not only by the RNA sequence and chemical modifications, but also by their replication competency, delivery vehicle, and route of administration. These factors determine the magnitude, duration, and type of host responses, including innate immunity, inflammation, and adaptive immune activation. While immune stimulation is desirable in some settings (e.g., vaccination and antiviral therapy), excessive or uncontrolled activation can lead to toxicity, impaired efficacy, or regulatory barriers. Understanding and managing these responses is essential for the safe clinical translation of both non-replicating and replicating RNA therapies.^2^^,^^134^

### Innate immune activation

Innate immune activation is a double-edged sword in RNA therapeutics: it can enhance efficacy in vaccines and antivirals but also pose barriers to safety and tolerability in therapeutic contexts. Host cells detect foreign RNA via PRRs, which trigger interferon responses and downstream inflammation. Key PRRs include the cytoplasmic sensors RLRs and endosomal TLRs.^11^^,^^124^^,^^125^

Non-replicating RNAs, such as conventional mRNAs used for protein replacement or vaccination, are typically engineered to minimize PRR activation. Strategies include incorporation of modified nucleosides (e.g., pseudouridine and 5-methylcytidine), use of optimized cap structures (e.g., CleanCap or anti-reverse cap analogs), and removal of double-stranded RNA contaminants during *in vitro* transcription.^22^^,^^90^^,^^135^ These design features help evade innate detection, especially important in chronic or systemic indications where repeated dosing is required.

Chemical modification is a cross-cutting strategy applied across all non-replicating RNA therapeutic classes, though the specific modifications and their purposes differ considerably by platform. For mRNA, N1mΨ has become the clinical standard for suppressing TLR7/8 and PKR activation while improving translational efficiency; codon optimization and cap analog selection further enhance protein output.^3^^,^^23^ For siRNAs, 2′-*O*-methyl and 2′-fluoro modifications at alternating positions of the guide and passenger strands reduce innate immune recognition, improve metabolic stability, and are now standard in all approved GalNAc-siRNA drugs; phosphorothioate backbone modifications at terminal positions further enhance nuclease resistance and tissue retention.^14^ For ASOs, the backbone and sugar chemistry define therapeutic behavior: phosphorothioate substitution throughout the backbone confers nuclease resistance and protein binding that extends plasma half-life, while 2′-*O*-methoxyethyl, LNA, or constrained ethyl (cEt) sugar modifications in the flanking wings of gapmer ASOs increase target affinity and allow dose reduction.^33^ For antimiRs, LNA modifications are particularly important because the very high target affinity they confer allows short oligonucleotide lengths (as few as 8 nucleotides) that retain potent miRNA inhibition while improving tissue distribution.^122^ For aptamers, 2′-fluoro and 2′-*O*-methyl pyrimidine substitutions are routinely incorporated during SELEX to generate nuclease-resistant candidates, and PEG conjugation extends renal clearance half-life from minutes to hours or days.^16^ Across all these platforms, the common goals of chemical modification are nuclease resistance, reduced innate immune stimulation, improved pharmacokinetics, and enhanced target affinity, but the chemical solutions are platform-specific and have been developed and refined over decades of independent optimization.

saRNAs encode viral replicase proteins (e.g., alphavirus nsP1-4) that enable intracellular RNA replication. This self-amplification boosts antigen expression and generates replication intermediates such as long double-stranded RNAs and 5′-triphosphorylated RNAs, potent agonists of RIG-I and MDA5.^11^^,^^88^^,^^123^^,^^124^ As a result, saRNAs frequently induce robust interferon responses.^67^^,^^91^ While this adjuvant-like effect can enhance vaccine efficacy,^136^ it may limit tolerability at higher doses or in non-vaccine settings.^137^ Furthermore, strong innate activation may inhibit translation of the payload protein through PKR-mediated pathways, adding a layer of complexity to therapeutic applications.^91^

DI RNAs, although replication-competent, are typically derived from viral genomes with large deletions. Most DI RNAs rely on structural features, 5′-triphosphate termini, and replicative intermediates to engage cytoplasmic PRRs, particularly RIG-I and MDA5, triggering potent interferon responses and antiviral effects.^129^ However, studies by Vignuzzi and colleagues have shown that certain DI RNAs, such as ZIKV-derived DVGs, can retain open reading frames and produce defective or truncated proteins, adding complexity to their immunogenic profiles.^70^ Furthermore, Carolina López’s work has demonstrated that DVGs robustly stimulate interferon responses and can shape adaptive immunity depending on their abundance and structure.^6^^,^^115^^,^^129^ Importantly, while DI RNAs have shown therapeutic benefit in numerous models, including for influenza, RSV, and DENV, safety considerations remain essential. Although generally well tolerated across multiple animal models, dose, formulation, and route of administration must be optimized to avoid excessive cytokine responses.^73^^,^^126^^,^^132^

The magnitude and character of innate immune activation are shaped not only by RNA type but also by delivery route, formulation, and intracellular trafficking. For example, the route of administration (e.g., intramuscular vs. intravenous) can influence tissue tropism and the extent of systemic exposure, impacting the profile and magnitude of cytokine responses.^2^ Delivery vehicles also play a key role. LNPs typically enter cells via endocytosis, exposing RNA to endosomal TLRs before cytoplasmic escape. In contrast, extracellular vesicles (EVs) may fuse with the plasma membrane or enter through alternate pathways, potentially delivering RNA more directly to the cytoplasm and favoring recognition by RLRs.^138^ These differences in intracellular routing can markedly alter the interferon profile, inflammatory cytokine release, and translational repression outcomes.

Thus, innate immune engagement varies markedly across RNA modalities. Non-replicating RNAs are designed to minimize activation of PRRs and allow repeated dosing, while replicating RNAs (saRNAs and DI RNAs) intentionally, or unavoidably, engage innate pathways to enhance antiviral potency or immunogenicity. A mechanistic understanding of PRR activation and its consequences, interferon production, and inflammatory cytokine release, is essential for optimizing both safety and therapeutic efficacy across indications.

While many RNA platforms seek to evade innate immune detection, some RNAs are instead engineered specifically to activate PRRs as their primary mechanism of action, functioning as RNA-based immunostimulants or adjuvants rather than as a separate therapeutic platform class. An illustrative example is CV8102 (CureVac), an intratumorally administered, non-coding RNA developed for cancer immunotherapy. CV8102 is engineered to activate endosomal TLR7/8 and cytoplasmic RIG-I, inducing local type I interferon production and pro-inflammatory cytokine release that enhance antigen presentation and anti-tumor immune responses.^139^ CV8102 has been evaluated in a Phase 1 clinical trial of intratumoral administration in patients with advanced melanoma, squamous cell carcinoma of the skin, squamous cell carcinoma of the head and neck, and adenoid cystic carcinoma, demonstrating a manageable safety profile and early signals of antitumor activity.^139^

This strategy exemplifies the use of synthetic RNA as a deliberate innate immune agonist, in which immunogenicity is not a liability but the therapeutic objective.

### Cytokine responses and systemic toxicity

Innate immune activation by RNA therapeutics frequently results in downstream production of pro-inflammatory cytokines, including interferons, interleukin-6, and tumor necrosis factor. While these responses are desirable in vaccine and antiviral contexts, excessive or dysregulated cytokine release can lead to systemic inflammatory symptoms and limit tolerability.^2^^,^^43^^,^^134^

At a mechanistic level, increasing RNA dose and intracellular persistence are associated with enhanced engagement of cytoplasmic and endosomal PRRs. This sensing drives interferon production and downstream cytokine cascades, linking RNA abundance and replication kinetics directly to innate immune activation.^2^^,^^62^^,^^67^^,^^124^^,^^125^

In both preclinical and early clinical development, RNA therapeutics, particularly saRNAs and unmodified mRNAs, have demonstrated dose-dependent innate immune activation. While such responses can enhance immunogenicity, excessive activation may manifest as transient systemic reactogenicity and acute-phase symptoms, necessitating careful balancing of potency and tolerability.^2^^,^^67^^,^^140^ Consequently, saRNA platform development has emphasized dose optimization, formulation refinement, and delivery strategies that localize RNA exposure or attenuate systemic innate immune signaling.^61^^,^^91^^,^^93^^,^^137^ Collectively, early translational experience underscores that controlling amplification-driven immune activation remains a central design constraint for replicating RNA modalities.

Clinically, innate immune responses to mRNA-LNP vaccines, such as those deployed for COVID-19, are commonly associated with transient flu-like symptoms, including fever, fatigue, myalgia, and headache. These effects are generally self-limiting and are attributed to interferon and cytokine induction following vaccination.^2^ Early human trials of saRNA vaccines from Imperial College London and Arcturus Therapeutics further demonstrated that interferon-mediated reactogenicity could become dose-limiting, prompting reformulation and dose-adjustment strategies to improve tolerability.^67^^,^^136^

Rare immune-mediated myocarditis has also been reported following administration of mRNA-based COVID-19 vaccines, predominantly after the second dose and most frequently in adolescent and young adult males.^141^^,^^142^ Clinical presentation is typically mild and the short-term clinical course is generally favorable, with no cardiac deaths or transplantations reported in a large longitudinal US multicenter cohort at a median follow-up of approximately six months.^143^ However, the same study found that late gadolinium enhancement on cardiac magnetic resonance imaging (MRI) persisted in 60% of patients at follow-up, indicating subclinical myocardial injury that warrants continued clinical surveillance and longer-term outcome data.^143^ Although the precise mechanism remains incompletely defined, current evidence supports an immune-mediated process, potentially involving transient innate immune activation, cytokine signaling, and antigen expression kinetics, rather than direct myocardial toxicity.^142^ Importantly, large population-based studies indicate that the incidence and severity of myocarditis following mRNA vaccination are substantially lower than those observed following SARS-CoV-2 infection itself.^144^ From a platform perspective, these findings reinforce the importance of balancing immune activation with tolerability as RNA technologies evolve toward longer intracellular persistence.

In addition to the RNA cargo itself, LNP delivery systems may contribute independently to systemic immune responses. Ionizable or cationic lipid components can engage innate immune receptors, including TLR2 and TLR4, and are implicated in inflammatory signaling.^140^ Preferential accumulation of LNPs in immune-rich organs such as the liver and spleen may further amplify cytokine responses, particularly when combined with replicating RNA species that prolong RNA exposure.^2^^,^^91^

DI RNAs often exhibit a more transient immune activation profile, reflecting their historically non-coding nature and reliance on co-delivered replicase or helper virus. Nevertheless, some DI RNAs retain partial coding sequences and can express truncated viral proteins, potentially influencing their immunogenic properties.^70^^,^^129^ Regardless of coding capacity, DI RNAs robustly activate innate sensors such as RIG-I and MDA5 through replication intermediates and structured RNA motifs. While this interferon induction underpins antiviral efficacy, tolerability remains dependent on dose, route of administration, and the context of infection.^72^^,^^126^

Finally, experience with recombinant interferon therapies, such as interferon-alpha (IFN-α) and interferon-beta (IFN-β) for viral hepatitis and multiple sclerosis, illustrates that sustained interferon signaling can lead to cytopenias, hepatic inflammation, and neuropsychiatric side effects.^145^ Although RNA therapeutics rely on endogenous interferon pathways rather than exogenous cytokine administration, these clinical precedents underscore the importance of rigorous preclinical toxicology and cautious dose escalation, particularly for intravenously delivered or highly immunostimulatory RNA constructs.

### Adaptive immune responses and Re-administration

The adaptive immune profile of RNA therapeutics is shaped indirectly by innate sensing, which drives antigen presentation and primes T and B cell responses. This phenomenon, often referred to as intrinsic adjuvanticity, is a key strength of RNA platforms, particularly in vaccine settings.^2^^,^^88^ Innate activation via RLRs and TLRs leads to upregulation of costimulatory molecules and proinflammatory cytokines, enhancing dendritic cell maturation and antigen-specific immunity.^125^

saRNAs are especially potent in this regard. Their replication leads to prolonged antigen expression and production of double-stranded RNA intermediates that serve as potent immunostimulants.^67^^,^^88^^,^^93^ Preclinical and clinical studies of saRNA vaccines have shown robust CD4^+^ and CD8^+^ T cell responses and long-lasting antibody titers.^136^^,^^137^ This immunogenicity, while beneficial in vaccine contexts, may pose challenges for repeat administration if neutralizing antibodies or T cell responses develop against vector components, especially when delivered via viral particles.^75^

In contrast, non-replicating mRNAs tend to induce less durable immunity unless formulated with immunostimulatory adjuvants or delivered in potent LNP systems.^2^^,^^23^^,^^145^ Re-administration is typically feasible, although PEGylated lipids in LNPs can elicit anti-PEG antibodies in some recipients, potentially impacting biodistribution or triggering hypersensitivity reactions on repeat dosing.^146^

For DI RNAs, adaptive responses are less well characterized. These constructs typically lack protein-coding capacity, although some retain truncated open reading frames and may express small or defective peptides.^70^ In theory, this limits the generation of anti-RNA or anti-protein antibodies. However, the strong interferon response elicited by DI RNAs may contribute to enhanced antigen presentation and adaptive priming when co-administered with viral antigens or used in conjunction with co-infecting viruses.

Repeated dosing of RNA therapeutics may also trigger host responses to non-RNA components, such as delivery vehicles (e.g., LNPs and EV membranes) or replicon-associated proteins. For example, alphavirus replicon RNAs or envelope proteins used in packaging can be immunogenic and may reduce transduction efficiency upon re-administration unless strategies such as pseudotyping or heterologous boosting are employed.^78^ By contrast, direct evidence on this point has recently emerged: in mice, an initial saRNA vaccination elicits replicase-specific antibody and T cell responses that reduce the *in vivo* expression and Th1 responses of a subsequently administered saRNA vaccine.^147^ Notably, however, this pre-existing anti-replicase immunity did not compromise the protective efficacy of a subsequent influenza saRNA vaccine against lethal H5N1 challenge,^147^ indicating that the impact of anti-replicase immunity on repeat dosing may be context- and readout-dependent.

In summary, RNA therapeutics, especially replicating forms, have inherent immunostimulatory properties that promote adaptive immune engagement. This self-adjuvant effect is advantageous in vaccines but must be carefully managed in therapeutic settings where repeated administration or prolonged expression is required.

## Controllability and off-target risks

Building on the immunostimulatory and adaptive immune properties discussed in the previous sections, an additional critical consideration for RNA therapeutics is the degree of control over RNA persistence, amplification, and off-target activity. One of the key advantages of non-replicating RNAs is their limited persistence and relatively predictable pharmacokinetics. These RNAs are typically degraded within hours to days, enabling precise temporal control of protein expression or immunomodulatory effects. Such controllability is particularly valuable in therapeutic settings requiring transient expression, low systemic exposure, or repeated dosing without cumulative toxicity.^2^

In contrast, replicating RNA platforms introduce additional complexity due to their capacity for intracellular amplification. Once inside target cells, these RNAs can generate high copy numbers of themselves and/or encoded products, extending both the magnitude and duration of biological effects. While this self-amplification reduces dosing requirements and can enhance efficacy, it also introduces greater uncertainty in the timing, intensity, and tissue distribution of RNA activity.^67^^,^^136^

For saRNAs, prolonged or ectopic expression of encoded proteins (e.g., antigens or cytokines) may increase the risk of off-target inflammation, cellular stress, or disruption of normal tissue function.^91^^,^^137^ These risks are exacerbated when delivery systems lack strict tissue targeting or when encoded products exert bioactivity across multiple cell types. In addition, alphavirus-derived replicase complexes used in saRNA systems exhibit a degree of template flexibility and have been shown in experimental systems to act in trans on RNAs containing compatible *cis*-acting replication elements. This raises a theoretical risk of unintended amplification of co-delivered or endogenous RNA species, although the extent of such effects in therapeutic settings remains incompletely defined.^88^^,^^101^

DI RNAs, by contrast, generally lack full protein-coding capacity and are therefore considered less prone to off-target translation effects. However, accumulating evidence indicates that some DI RNAs retain truncated open reading frames capable of producing aberrant or immunogenic peptides, adding complexity to their safety profile.^6^^,^^70^ Furthermore, because DI RNAs depend on a co-infecting virus for replication, the presence of latent, persistent, or undetected viral infections, particularly in immunocompromised individuals, raises the possibility of unintended amplification or recombination events.^126^^,^^132^

Recombination represents a broader concern across replicating RNA technologies. In replicon RNAs derived from pathogenic viruses or constructs containing repetitive or homologous elements, recombination with viral genomes or, less commonly, host transcripts could theoretically reconstitute replication-competent viruses or generate novel variants with altered pathogenicity.^115^^,^^124^ Regulatory agencies have therefore identified recombination risk as a priority safety consideration, motivating design strategies that minimize sequence homology, employ non-pathogenic backbones, or incorporate safeguards such as deletion of essential replication elements.^129^

To mitigate these risks, comprehensive preclinical evaluation is therefore essential to assess biodistribution, persistence, and unintended protein expression across tissues.^134^ Emerging RNA design strategies, including incorporation of miRNA target sites for tissue-restricted degradation, synthetic kill-switch circuits, and inducible or self-limiting replicase systems, offer promising approaches for achieving tighter spatiotemporal control of replicating RNA activity. Such innovations are likely to be critical for safely deploying replicating RNA therapeutics beyond vaccine applications.^79^^,^^100^

## Conclusion

RNA therapeutics have reshaped the landscape of modern medicine, offering programmable and adaptable tools to modulate gene expression, deliver antigens, and suppress pathogens.^2^^,^^23^ Within this rapidly advancing field, the distinction between non-replicating and replicating RNA modalities provides a useful framework for understanding how molecular design influences therapeutic behavior, delivery strategies, safety profiles, and clinical applications.^91^^,^^124^

Non-replicating RNAs, such as siRNAs, ASOs, and mRNAs, have achieved clinical success by enabling controllable, transient effects through increasingly sophisticated delivery technologies.^22^ Their defined mechanisms of action and a maturing regulatory environment have positioned them at the forefront of approved RNA-based medicines, including treatments for genetic diseases and vaccines for emerging infections.^23^^,^^134^

In contrast, replicating RNAs, including saRNAs and DI RNAs, offer transformative advantages in potency, duration, and innate immune activation.^67^^,^^72^^,^^88^ These features make them especially attractive for vaccines, antiviral therapies, and field-deployable applications. These advances are complemented by inherent dose-sparing potential and improved manufacturability, which together will be critical determinants of clinical and global deployment. However, their complexity, immunogenicity, and potential off-target effects pose distinct translational challenges that must be addressed through rigorous design and testing.^129^^,^^137^ Among replicating platforms, DI RNAs occupy a distinctive position by decoupling intracellular RNA amplification from productive infection, offering broad antiviral activity with emerging preclinical evidence for favorable safety and self-limiting behavior.

Importantly, these categories are not mutually exclusive. Advances in synthetic biology and RNA engineering are enabling hybrid platforms that combine amplification with precision and immune stimulation with enhanced safety.^79^^,^^100^ Delivery innovations aimed at improving RNA stability and manufacturability are also expanding the translational potential of RNA therapeutics. For example, lyophilization strategies have been shown to preserve the stability and antiviral activity of DIPs, enabling long-term storage and reducing dependence on cold-chain logistics. Such advances are especially relevant for field-deployable antiviral countermeasures and pandemic preparedness, where stability, scalability, and distribution are critical determinants of real-world impact.^148^

Circular RNA (circRNA) represents a further emerging format that does not fit neatly into either the replicating or non-replicating categories as defined here, but merits brief mention as a platform whose trajectory will intersect with both. circRNAs are covalently closed, single-stranded RNA molecules that lack 5′ caps and poly(A) tails, rendering them resistant to exonucleolytic degradation and conferring substantially greater intracellular stability than linear mRNA.^149^ This stability advantage is attractive for sustained protein expression or gene silencing applications without the need for intracellular amplification. However, the manufacturing of clinical-grade circRNA is complicated by the presence of linear RNA impurities arising from incomplete circularization during *in vitro* transcription; recent work has characterized the immunostimulatory potential of these impurities and underscored the importance of purification strategies analogous to those used to remove dsRNA from conventional mRNA preparations.^150^ As circularization chemistry, purification methods, and delivery formulations mature, circRNA is likely to emerge as a distinct non-replicating platform with a unique pharmacokinetic profile, complementing rather than replacing existing linear RNA modalities.

As the field matures, the future success of RNA therapeutics will hinge not only on scientific breakthroughs but also on integrating safety, manufacturability, and regulatory considerations into development pipelines. Whether for pandemic preparedness, cancer immunotherapy, or rare genetic disorders, RNA medicines are evolving into a scalable and durable platform. By explicitly comparing replicating and non-replicating RNA strategies, and understanding their trade-offs and synergies, we can more rationally design the next generation of RNA therapeutics that are safe, adaptable, and clinically impactful.

## Acknowledgments

This work was supported by the 10.13039/501100000925National Health and Medical Research Council of Australia through Ideas Grant 2011398. The authors thank colleagues and collaborators who contributed discussions and feedback during the development of the manuscript. Some icon elements in the graphical abstract are obtained from Lab icons (https://lab-icons.com).

## Author contributions

Conceptualization, M-H.L. and D.H.; writing – original draft, review and editing, M-H.L. and D.H.

## Declaration of interests

The authors declare no competing interests.

## Declaration of generative AI and AI-assisted technologies in the manuscript preparation process

During the preparation of this work, the authors used ChatGPT (OpenAI) to assist with editorial review, language refinement, and reference checking. Following the use of this tool, the authors reviewed and edited the content as appropriate and take full responsibility for the content of the published article.
